# Impact of APOE ε4 on Cognitive Progression in Individuals with Subjective Cognitive Decline: Preliminary Data from a Brazilian Cohort

**DOI:** 10.1002/alz70857_107228

**Published:** 2025-12-25

**Authors:** Bruno De Oliveira De Marchi, Barbara Loeblein Uebel, Victória Tizeli Souza, Simone Sieben da Mota, Gabriela Raquel Paz Rivas, Guilherme Da Silva Carvalho, Haniel Bispo De Souza, Lucas Bastos Beltrami, Rhaná Carolina Santos, Sarah Vitoria Bristot Carnevalli, Ana Letícia Amorim de Albuquerque, Leonardo Martins de Paula, Manuella Edler Zandoná Giordani, Wyllians Vendramini Borelli, Marcia L. Fagundes Chaves, Eduardo R. Zimmer, Raphael Machado Castilhos

**Affiliations:** ^1^ Universidade Federal do Rio Grande do Sul, Porto Alegre, RS, Brazil; ^2^ Hospital de Clínicas de Porto Alegre, Porto Alegre, RS, Brazil; ^3^ Hospital de Clínicas de Porto Alegre, Porto Alegre, Rio Grande do Sul, Brazil; ^4^ Universidade do Vale do Rio dos Sinos, São Leopoldo, Rio Grande do Sul, Brazil; ^5^ Universidade Federal de Santa Catarina, Florianópolis, Santa Catarina, Brazil; ^6^ Universidade Federal do Rio Grande do Sul, Porto Alegre, Rio Grande do Sul, Brazil; ^7^ Centro de Memória, Hospital Moinhos de Vento, Porto Alegre, RS, Brazil; ^8^ Masima: Macunaíma Soluções em Imagens Médicas, Porto Alegre, Rio Grande do Sul, Brazil; ^9^ Clinical Hospital of Porto Alegre, Porto Alegre, Rio Grande do Sul, Brazil; ^10^ Hospital Moinhos de Vento, Porto Alegre, Rio Grande do Sul, Brazil; ^11^ McGill Centre for Studies in Aging, Montreal, QC, Canada; ^12^ Brain Institute of Rio Grande do Sul (InsCer), PUCRS, Porto Alegre, Rio Grande do Sul, Brazil

## Abstract

**Background:**

**Subjective cognitive decline (SCD) and apolipoprotein E (*APOE*) ε4 allele are known risk factors for Alzheimer's disease (AD). However, the impact of *APOE* ε4 on cognitive progression in individuals with SCD remains unclear. The aim of this study is to evaluate whether SCD *APOE* ε4 carriers show greater cognitive decline over one year compared to non‐carriers**.

**Method:**

We used data from the BRASCODE cohort, including 142 participants with SCD divided into *APOE* ε4 carriers (ε4+) and non‐carriers (ε4−). Cognitive function was assessed using the Memory Complaint Scale (MCS) (both self‐reported and informant‐based), the Subjective Cognitive Decline Scale (SCD‐S), the Mini Mental State Examination (MMSE), the Clinical Dementia Rating (CDR) Scale, the Geriatric Anxiety Inventory (GAI) and the Geriatric Depression Scale (GDS). We applied linear mixed models using the baseline and the one year follow‐up data to evaluate the interaction between time and *APOE* ε4 in the cognitive progression of SCD patients.

**Result:**

In the ε4− group, there were a total number of 97 participants (73.2% females), with a median age of 69 years [67, 72] and 16 years of formal education [11, 18]. In the ε4+ group, there were 42 participants (64.3% females), with a median age of 71 [67.25, 75] and 15 years of formal education [11, 17]. There was no statistically significant difference between the groups in cognitive test outcomes, sleep disturbances, anxiety, depression, or the global Z score. However, a tendency to higher prevalence of family history was observed in the ε4+ group (61.9%) compared to ε4− (42.3%), *p* = 0.052. Findings from the linear mixed model indicated that ε4+ was not a significant predictor of cognitive decline in SCD patients over one year, *p* = 0.217.

**Conclusion:**

Even though *APOE* ε4 is a well established risk factor for the development of AD, our findings suggest that the presence of this allele was not associated with significant impact on cognitive progression over a one‐year period in individuals with SCD. This suggests that factors other than *APOE* ε4 may influence SCD trajectories, and longer follow‐up studies are needed to clarify this relationship.

